# Synthesis of Silicon Dioxide (SiO_2_) Nanowires via a Polyethylene Glycol-Based Emulsion Template Method in Isopropanol

**DOI:** 10.3390/nano15050326

**Published:** 2025-02-20

**Authors:** Jian Liu, Yonghua Sun, Tianfeng Yang

**Affiliations:** 1 Zhejiang Fuli Analytical Instrument Co., Ltd., Wenling 317500, China; yangtf@cnfuli.com.cn; 2 College of Materials and Chemistry & Chemical Engineering, Chengdu University of Technology, Chengdu 610059, China; sunyh@ruolin-lab.com

**Keywords:** SiO_2_ nanowires, polyethylene glycol, isopropanol, emulsion template

## Abstract

Typical wet-chemical methods for the preparation of silica nanowires use polyvinylpyrrolidone and n-pentanol. This study presents a polyethylene glycol-based emulsion template method for the synthesis of SiO_2_ nanowires (SiO_2_NWs) in isopropanol. By systematically optimizing key parameters (type of solvent, polyethylene glycol molecular weight and dosage, dosage of sodium citrate, ammonium and tetraethyl orthosilicate, incubation temperature and time), SiO_2_NWs with diameters about 530 nm were obtained. Replacing polyvinylpyrrolidone with polyethylene glycol enabled anisotropic growth in isopropanol, overcoming the dependency on traditional solvents like n-pentanol. Scale-up experiments (10× volume) demonstrated robust reproducibility, yielding nanowires with consistent morphology (~580 nm diameter). After calcination at 500 °C for 1 h, the morphology of the nanowires did not change significantly.

## 1. Introduction

SiO_2_ nanowires (SiO_2_NWs), characterized by their high aspect ratios and tunable diameters at the nanoscale [1,2,3], have emerged as an important class of one-dimensional nanomaterials with broad applications in photoluminescence [4,5], electronics [6,7,8], microwave devices [9,10], RO filtration [11], preparation of new materials [12], and so on. However, the realization of these applications depends on the precise control of their morphology, dimensions and structural uniformity during synthesis, which remains a major challenge. Many methods have been developed for the growth of SiO_2_NWs, such as chemical vapor deposition (CVD) [13,14], electrospinning [15,16], hydrothermal method [17,18], pulsed laser deposition [19], the vapor–liquid–solid method [20], thermal evaporation method [21], etc.

The template method is an important method for the synthesis of nanomaterials and has been widely used for the preparation of various materials, including SiO_2_NWs [22,23]. Template-based approaches using hard templates such as anodic aluminum oxide (AAO) [24] or soft templates [25,26,27] offer improved control over nanowire dimensions. However, hard templates necessitate post-synthesis removal steps that may compromise structural integrity, while soft templates often face limitations in stabilizing elongated nanostructures. These challenges highlight the need for innovative synthesis strategies that combine scalability, precision, and environmental benignity.

Emulsion templating has recently gained traction as a versatile route for the fabrication of anisotropic silica nanostructures [28,29]. The major breakthrough in this field resulted after Zhang et al. synthesized the wires using a polyvinylpyrrolidone (PVP)–pentanol–sodium citrate emulsion droplet system with gold particles as a soft templates [30]. Further investigation by Kuijk et al. [31] found that it is the sodium citrate, either coated on the Au particles or unbound, that aids in wire growth, and SiO_2_NWs can be synthesized in a PVP–pentanol–sodium citrate emulsion droplet system rather than using Au nanoparticles. The subtlety of this system is that it creates an environment in which TEOS can be unilaterally supplied.

Although many studies have demonstrated the feasibility of this system for the synthesis of SiO_2_NWs [7,32,33,34,35,36], challenges remain in the growth of SiO_2_NWs in other non-amyl alcohols and non-PVP systems. In this study, we present a method for the synthesis of SiO_2_NWs using a PEG-based emulsion template in isopropanol as a continuous phase. Through a systematic investigation, we show how changing the synthesis parameters affects the morphology of SiO_2_NWs. Through an optimized methodology, SiO_2_NWs (approximately 530 nm in diameter) can be reliably synthesized. A 10-fold expansion of this process still yielded stable wires with diameters of about 580 nm. We hope that this work provides a new route to SiO_2_NWs.

## 2. Materials and Methods

### 2.1. Materials

Tetraethyl orthosilicate (TEOS, Chengdu Kelong Chemical Co., Ltd., Chengdu, China), ammonia (25–28%, Chengdu Kelong Chemical Co., Ltd.), polyethylene glycol (PEG, MW = 300, 600, 6000 and 10,000 g/mol); Shanghai Macklin Biochemical Technology Co., Ltd., Shanghai, China) and sodium citrate (Shanghai Aladdin Chemistry Co., Ltd., Shanghai, China) were all used as received without any purification. Deionized water with a resistivity of 18.0 MΩ·cm was used in all the experiments.

### 2.2. Preparation of SiO_2_NWs

A typical process obtained after process optimization is as follows.

Weigh 0.5 g of PEG (molecular weight: 6000 g/mol) and transfer it into a 10 mL plastic centrifuge tube. Add 5 mL of isopropanol and subject the mixture to sonication until the PEG is completely dissolved. Then, add the following reagents in sequence, with brief manual agitation after each addition: 325 µL of water, 300 µL of 0.05 M sodium citrate, and 75 µL of ammonium. After the addition of these components, add 300 µL of tetraethyl orthosilicate (TEOS) to the reaction mixture with thorough mixing. The reaction vessel should then be transferred to a temperature-controlled water bath maintained at 75 °C for a duration of 4 h to facilitate the synthesis process. Upon completion of the reaction, the resulting product should be isolated by centrifugation (4000 rmp). Then, wash the material obtained five times with ethanol and water and then dry at 80 °C for 6 h to obtain the final product.

### 2.3. Scale-Up Experiment

Weigh 1.5 g of PEG (molecular weight: 6000 g/mol) and transfer it into a 50 mL glass flask. Add 50 mL of isopropanol and sonicate until the PEG is completely dissolved. Then, add the following reagents in sequence, with a brief manual agitation after each addition: 3.25 mL of water, 3 mL of 0.05 M sodium citrate, and 0.75 mL of ammonium. Following the addition of these components, incorporate 3 mL µL of TEOS into the reaction mixture with thorough mixing. The glass flask should then be transferred to a temperature-controlled water bath maintained at 75 °C for a duration of 4 h to facilitate the synthesis process. Upon completion of the reaction, the resulting product should then isolated by centrifugation (4000 rmp). Then, was the obtained material five times with ethanol and water and dry at 80 °C for 6 h to yield the final product.

### 2.4. Characterization

The morphology of the synthesized samples was examined using a Field Emission Scanning Electron Microscope (FE-SEM, FEI Quattro S, USA). The specific surface area and pore size distribution were characterized using a specific surface area and pore size analyzer (BSD-PM1/2, Best Instruments, Inc., Dongguan, China). The surface chemical functional groups were identified through Fourier Transform Infrared Spectroscopy (FTIR, IS5 Nicolet, Thermo Fisher Scientific Inc., USA).

## 3. Results and Discussion

### 3.1. Effect of Solvent

**Figure 1:**
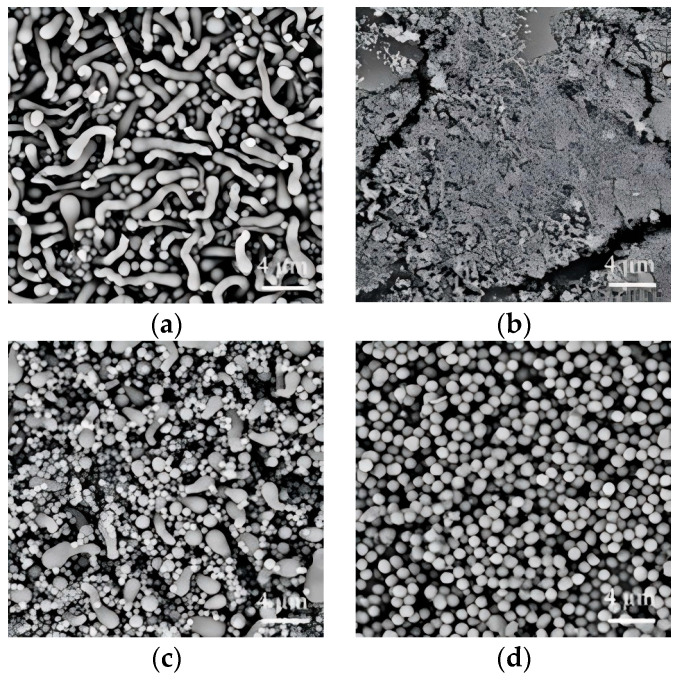
SiO_2_NWs prepared in isopropyl alcohol (**a**), ethanol (**b**), N-propyl alcohol (**c**), N-pentyl alcohol (**d**). (0.05 g PEG, 200 μL sodium citrate, 50 μL ammonium, 300 μL TEOS).

To our knowledge, n-pentanol has been predominantly employed as the solvent of choice the synthesis of SiO_2_NWs by wet-chemical methods, with limited exploration of alternative solvent systems. In this investigation, we examined the solvent effect on nanowire morphology by employing isopropanol, ethanol, n-propanol, and n-pentanol as the reaction media. Our results reveal a pronounced solvent-dependent morphological evolution of the silica nanostructures. As illustrated in Figure 1, the solvent selection significantly influenced the resulting nanostructure morphology. Isopropanol-mediated synthesis yielded predominantly curved, tadpole-like architectures with an average diameter of 830 nm, accompanied by spherical particles (Figure 1a). Ethanol as the reaction medium produced finely granular structures (Figure 1b), while n-propanol generated a mixture of spherical particles and a minor fraction of tadpole-like morphologies (Figure 1c). Notably, n-pentanol exclusively produced spherical nanoparticles with an average diameter of 950 nm (Figure 1d), demonstrating the critical role of solvent selection in morphological control.

### 3.2. Effect of Molecular Weight and Dosage of PEG

**Figure 2:**
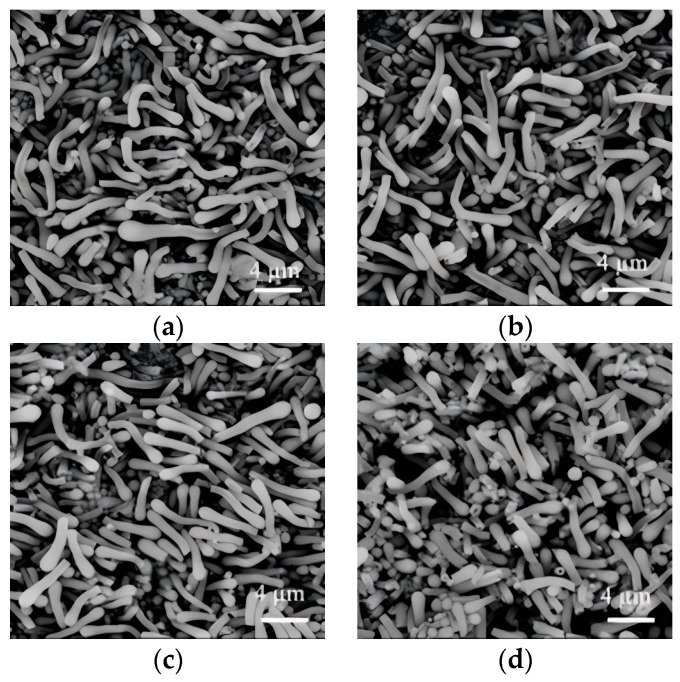
SiO_2_NWs synthesized using PEGs with molecular weight of 300 g/mol (**a**), 600 g/mol (**b**), 6000 g/mol (**c**), and 10,000 g/mol (**d**); (0.05 g PEG, 200 μL sodium citrate, 50 μL ammonium, 300 μL TEOS).

In the conventional wet-chemical synthesis of SiO_2_NWs, PVP has been predominantly employed as an additive, while the application of PEG remains unreported in the literature. This study presented an investigation into the morphological control of SiO_2_NWs using PEG with varying molecular weights (300 g/mol, 600 g/mol, 6000 g/mol, and 10,000 g/mol). As demonstrated in Figure 2, the rod-like products were obtained when PEG with molecular weights of 300, 600 and 6000 were employed, with average diameters about 650 nm, 680 nm, and 620 nm (Figure 2a–c). However, the morphological uniformity was compromised when higher-molecular-weight PEG (10,000 g/mol) was used, resulting in a mixed-phase product consisting of short rod-like structures (about 710 nm in diameter) accompanied by particulate formations (Figure 2d).

**Figure 3:**
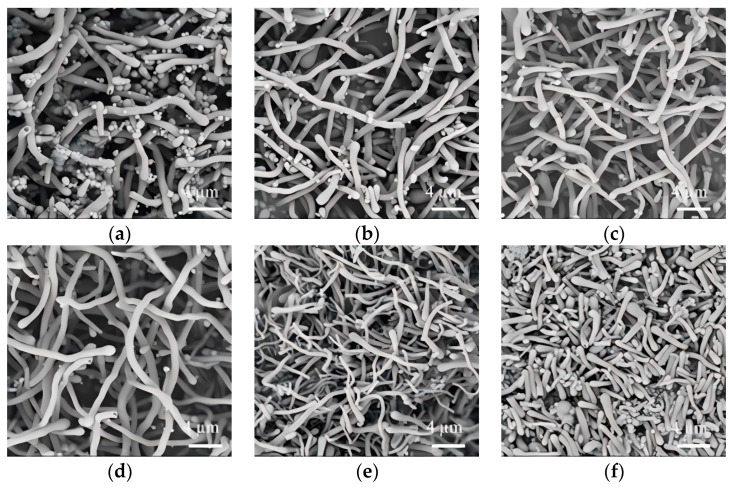
Effect of PEG dosage (molecular weight: 6000 g/mol) on the morphology of SiO_2_NWs. ((**a**), 0 g; (**b**), 0.01 g; (**c**), 0.05 g; (**d**), 0.5 g; (**e**), 0.7 g; (**f**), 1 g); (300 μL sodium citrate, 75 μL ammonium, 300 μL TEOS).

The morphological dependence of SiO_2_NWs on PEG dosage was also systematically investigated, as depicted in Figure 3. In the control experiment without PEG addition, the product exhibited a heterogeneous mixture of both nanowires and irregular nanoparticles (Figure 3a). Upon the introduction of PEG at dosages of 0.01–0.5 g, a morphological uniformity was achieved, with the formation of continuous nanowires exhibiting high aspect ratios (Figure 3b–d). However, beyond a dosage of 0.7 g, a significant morphological transition occurred, resulting in thinner and shorter nanowires (Figure 3e). Further increasing the PEG dosage to 1.0 g induced a complete morphological transformation, yielding discrete nanorods with an average diameter of 630 nm (Figure 3f).

### 3.3. Effect of Sodium Citrate Dosage

**Figure 4:**
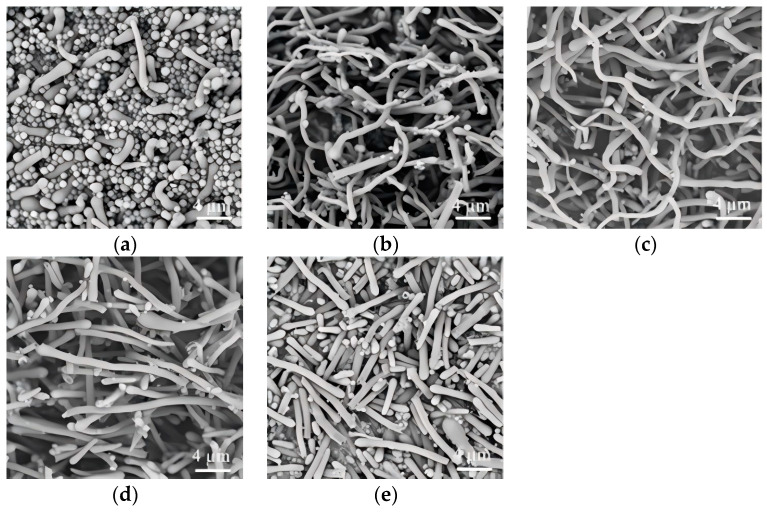
Effect of sodium citrate dosage (µL, 0.05 M) on the morphology of SiO_2_NWs. ((**a**), 100 µL; (**b**), 200 µL; (**c**), 300 µL; (**d**), 450 µL; (**e**), 650 µL); (0.05 g PEG, 50 μL ammonium, 300 μL TEOS).

The morphological evolution of silica nanostructures demonstrated a strong dependence on the concentration of sodium citrateon, which functions as a nucleating agent due to its limited solubility in isopropanol. When introduced into the isopropanol, sodium citrate forms particulate seeds that direct the growth of the nanowires. Systematic investigations revealed that the sodium citrate dosage significantly influences the final morphology within the investigated dosage range (100–650 µL). At the lower dosage (100 µL, Figure 4a), the product consisted of tadpole-shaped nanorods accompanied by a significant amount of spherical nanoparticles, suggesting incomplete morphological control. Moderate doses (200–450 µL, Figure 4b–d) resulted in curved nanowires, indicating enhanced growth directionality. Notably, at the highest dosage (650 µL, Figure 4e), the morphology transitioned to straight nanorods with reduced aspect ratios, demonstrating the critical role of sodium citrate in modulating both the curvature and dimensional characteristics of the nanostructures. At low sodium citrate dosages, fewer seed particles are formed, and the amount of TEOS is in excess relative to these seed particles. As a result, a large amount of TEOS is hydrolyzed and polymerized in isopropanol, leading to the formation of spherical particles. Conversely, when the concentration of sodium citrate is high, there is insufficient TEOS relative to the available seed particles. Although this results in a sufficient number of seed particles in solution, the shortage of TEOS ultimately leads to the formation of numerous short rod-like structures.

### 3.4. Effect of Ammonia Dosage

**Figure 5:**
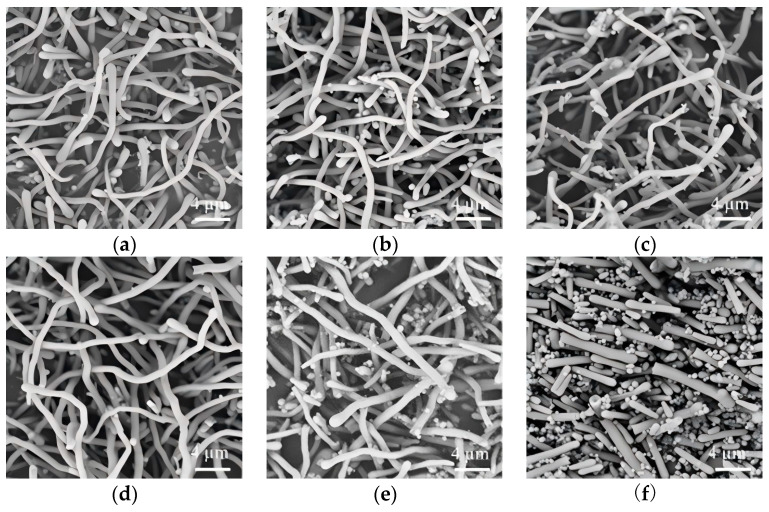
Effect of ammonia on the morphology of SiO_2_NWs. ((**a**), 10 µL; (**b**), 25 µL; (**c**), 50 µL; (**d**), 75 µL; (**e**), 100 µL; (**f**), 200 µL); (0.05 g PEG, 300 μL sodium citrate, 300 μL TEOS).

The morphological characteristics of SiO_2_NWs were found to be dependent on ammonia dosage, which governs the kinetics of TEOS hydrolysis and subsequent condensation reactions. A systematic investigation was carried out over an ammonia dosage range of 10–200 µL to elucidate its impact on nanowire growth. At a dosage in the range of 10–75 µL, wires with spherical termini were consistently observed (Figure 5a–d). As evidenced by previous reports [35,36], these characteristic bulbous ends exhibit sodium citrate encapsulation. Optimal morphology was achieved at 75 µL, yielding more homogeneous nanowires with diameters of about 630 nm (Figure 5d). Further increasing the ammonia dosage to 100 µL resulted in straighter nanowires (Figure 5e). However, at the highest dosage (200 µL), the products formed were straight, short rods along with a large number of particles (Figure 5f). This may be due to the fact that a large amount of ammonia causes more TEOS to be hydrolyzed and condensed in isopropanol, forming a large number of spherical particles instead of nanowires.

### 3.5. Effect of TEOS Dosage

**Figure 6:**
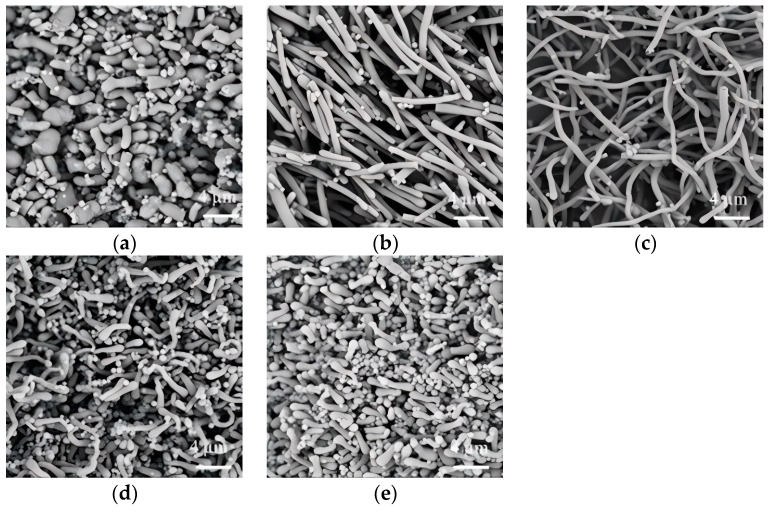
Effect of TEOS on morphology of SiO_2_NWs. ((**a**), 100 μL; (**b**), 200 μL; (**c**), 300 μL; (**d**), 400 μL; (**e**), 500 μL); (0.5 g PEG, 300 μL sodium citrate, 75 μL ammonium).

The morphological evolution of the silica nanostructures demonstrated significant sensitivity to TEOS dosage during the synthetic process. A systematic investigation over the dosage range of 100–500 μL in 5 mL isopropanol revealed distinct growth regimes (Figure 6). At a lower TEOS dosage (100 μL), the system exhibited incomplete nanowires development, yielding ellipsoidal particles accompanied by colloidal nanoparticles (Figure 6a). When increasing TEOS to 200 μL, well-defined straight nanorods with uniform diameters (640 nm, Figure 6b) were formed. Further elevation of TEOS to 300 μL induced morphological transition to curved nanowires with diameters of 540 nm (Figure 6c). Notably, exceeding the critical threshold of 400 μL resulted in complete morphological breakdown, characterized by spherical aggregates, due to precursor oversaturation initiating the direct condensation and isotropic growth in isopropanol rather than sodium citrate-mediated anisotropic growth (Figure 6d,e).

### 3.6. Effect of Incubation Temperature and Time

**Figure 7:**
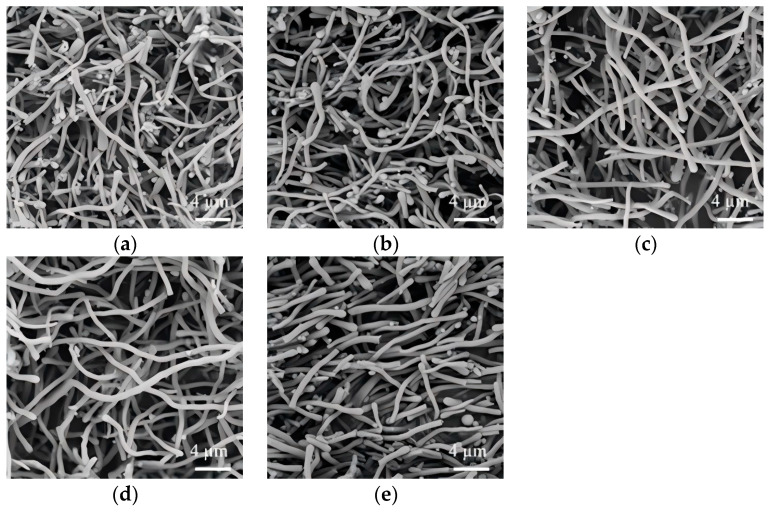
Effect of incubation temperature on the morphology of SiO_2_NWs. ((**a**), 25 °C; (**b**), 45 °C; (**c**), 65 °C; (**d**), 75 °C; (**e**), 80 °C); (0.5 g PEG, 300 μL sodium citrate, 75 μL ammonium, 300 μL TEOS).

Temperature-dependent variations primarily manifest in two aspects: (1) the hydrolysis/condensation reaction rates of TEOS molecules and (2) the diffusion kinetics of TEOS and its initial hydrolytic intermediates towards emulsion interfaces. As demonstrated by experimental observations, suboptimal thermal conditions (25–45 °C, Figure 7a,b) lead to heterogeneous nanostructures containing spherical particulate impurities. This morphological imperfection may be due to compromised mass transfer efficiency at lower temperatures, where delayed transportation of hydrolyzed silicon species to emulsion droplets permits premature polycondensation within the isopropanol continuous phase. Thermal optimization to 65–75 °C achieves monodisperse nanowire formation with uniform diameter distribution (530 nm, Figure 7c,d). Interestingly, excessive thermal input (80 °C) induces morphological transition to shortened rod-like structures with reduced diameters (500 nm, Figure 7e).

**Figure 8:**
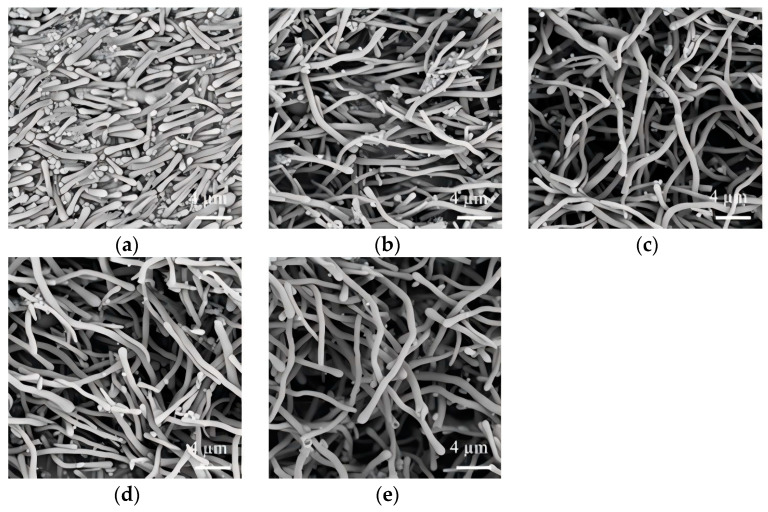
Effect of incubation time on morphology of SiO_2_NWs. ((**a**), 1 h; (**b**), 2 h; (**c**), 4 h; (**d**), 5 h; (**e**), 7 h); (0.5 g PEG, 300 μL sodium citrate, 75 μL ammonium, 300 μL TEOS).

Figure 8 illustrates how incubation time affects the morphology of the nanowires. At 1 h, a short rod with a larger head is formed (Figure 8a). Incubation times of 2, 4, 5 and 7 h produce well-defined nanowires (Figure 8b–e), although a few spherical particles appear at 2 and 4 h. After 7 h of incubation, the average diameter of the nanowires increases slightly to 570 nm (Figure 8e). This may be due to Oswald ripening.

### 3.7. Calcination of Nanowires and Product from Scale-Up Experiment

**Figure 9:**
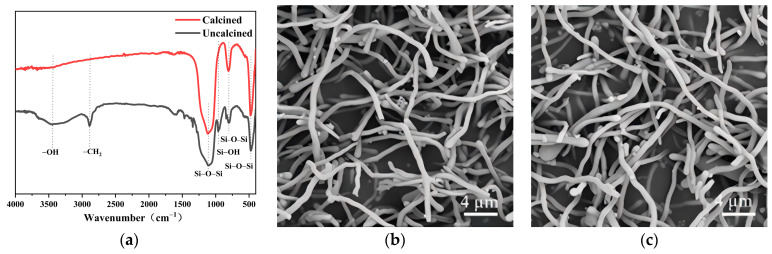
Changes in infrared spectra (**a**) and morphology before (**b**) and after (**c**) calcination (500 °C, 1 h).

SiO_2_NWs were prepared under optimized conditions, and the obtained nanowires were calcined at a high temperature to remove organic matter from them (500 °C, 1 h). IR spectra showed the presence of -OH (telescopic vibration: 3457.7 cm^−1^; antisymmetric telescopic vibration: 962.3 cm^−1^), -CH_2_ from PEG (2886.9 cm^−1^) and Si-O-Si (antisymmetric telescopic vibration: 1118.5 cm^−1^; bending vibration: 806.1 cm^−1^; rocking vibration: 474.4 cm^−1^). The IR spectra of the calcined nanowires show that only Si-O-S bonds are present in the product, while the -OH, -CH_2_, and Si-OH peaks disappear. This indicates that the product is mainly composed of SiO_2_, in which the organic matter have been removed. When calcined at 500 °C for 1 h, the morphology of the nanowires did not change significantly and remained intact as before (Figure 9b,c).

**Figure 10:**
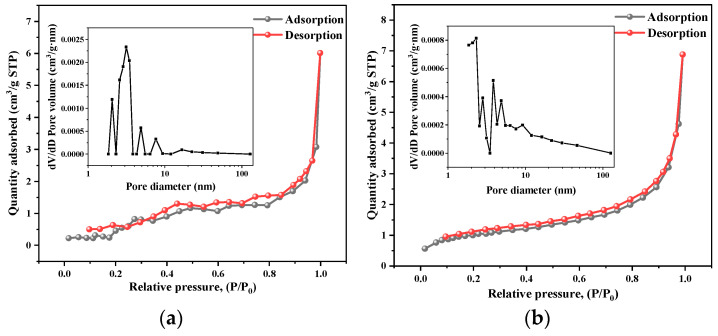
N_2_ adsorption and desorption curves and pore size distribution of SiO_2_NWs before (**a**) and after (**b**) calcination (500 °C, 1 h).

The Brunauer–Emmett–Teller method was used to determine the specific surface area of SiO_2_NWs before and after calcination and the Barrett–Joyner–Halenda (BJH) model was used to calculate the pore size distribution and pore volume. As shown in Figure 10a,b, all isotherms show a small hysteresis between adsorption and desorption, and type IV isotherm hysteresis curve [37]. The hysteresis loop of the curves shows the presence of the mesoporous and a small number of micropore structures in the materials. The specific surface area of the wires without calcination is 4.04 m^2^/g, while the specific surface area decreases to 3.09 m^2^/g after calcination.

**Figure 11:**
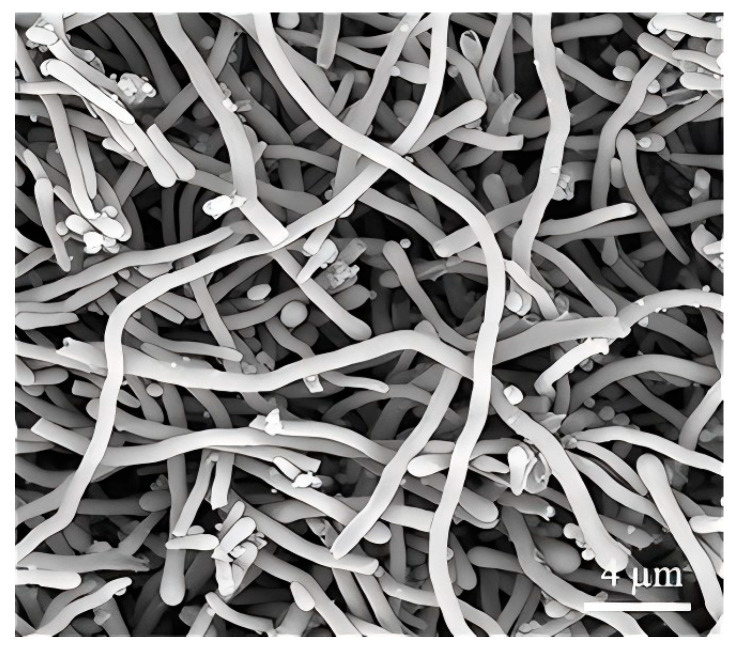
Electron microscope image of product from the scale-up experiment.

Under the optimized conditions, a 10-fold expansion of this process, with other reagents in the same proportions, as detailed in Section 2, still produced wires with average diameter about 580 nm (Figure 11) and good reproducibility.

## 4. Conclusions

In this study, a PEG-based emulsion template method for the synthesis of SiO_2_NWs in isopropanol was successfully developed, addressing the limitations of conventional template approaches. By systematically optimizing key parameters, typical SiO_2_NWs with uniform diameters (~530 nm) could be prepared in 5 mL of isopropanol by using 0.5 g of PEG (molecular weight: 6000 g/mol), 325 µL of water, 300 µL of 0.05 M sodium citrate, 75 µL of ammonium and 300 µL of TEOS, followed by heating at 75 °C for 4 h. After calcination at 500 °C for 1 h, the organic matter introduced during the preparation process was almost completely removed and the morphology of the nanowires did not change significantly. Notably, replacing traditional PVP with PEG expanded the scope of emulsion-templated synthesis, while the use of isopropanol as a continuous phase showed compatibility with the anisotropic growth of wires.

## Data Availability

The original contributions presented in this study are included in the article; further inquiries can be directed to the corresponding authors.
